# Genetically divergent methicillin-resistant *Staphylococcus aureus *and *sec*-dependent mastitis of dairy goats in Taiwan

**DOI:** 10.1186/1746-6148-8-39

**Published:** 2012-03-29

**Authors:** Chishih Chu, Changyou Yu, Yanhaui Lee, Yaochi Su

**Affiliations:** 1 Department of Microbiology, Immunology, and Biopharmaceuticals, National Chiayi University, No. 300, University Road, Chiayi 60004, Taiwan; 2 Department of Veterinary Medicine, National Chiayi University, No. 580, Sing Ming Road, Chiayi 60004, Taiwan

**Keywords:** MRSA, *Staphylococcus aureus*, Mastitis, Drug-resistance, MDR, Staphylococcal enterotoxin, Goats

## Abstract

**Background:**

Widespread in the environment, *Staphylococcus *spp. infect animals and humans as normal flora or pathogens. By extending our recent report of multi-drug resistant (MDR) *S. aureus *in dairy goats, this study investigated the staphylococcal infection and characterized the MDR-*S. aureus *and methicillin-resistant *S. aureus *(MRSA) isolates collected from goats in 2008 to elucidate the appearance of MRSA in goats and the mastitis associated staphylococcus enterotoxin (SE) types. A total of 555 samples were collected from six goat parts and three environmental sources among four dairy goat farms in southern Taiwan. Coagulase-positive and negative *Staphylococcus *spp. (CPS and CNS, respectively) were also identified. Furthermore, predominant SE genes of nine enterotoxin genes *sea *through *sej *along with antimicrobial resistance and genetic variations were determined.

**Results:**

In total, 137 staphylococcal strains were identified and found predominantly in milk, and in the vagina, anus, and nasal cavity. The most prevalent species was *S. lentus*, followed by *S. aureus, S. epidermidis*, and *S. xylosus*. Enterotoxin genes were not identified in any CNS isolates, however *sec *and *see *were identified only in *S. aureus *associated with mastitis in goat. In compared to the isolates from 2006 to 2007, 27 *S. aureus *isolates from 2008 were found to be more resistant to ampicillin, cephalothin, oxacillin, oxytetracycline, penicillin G, and tetracycline. Eleven MRSA isolates were identified and belonged to SCC*mec *type III (nine isolates) as the major type and SCC*mec *type II (two isolates). These MRSA isolates revealed pulse-field gel electrophoresis (PFGE) pattern A (five isolates), C (one isolate), and D (one isolate) of human isolates. The other two isolates without pulsotypes belonged to ST59.

**Conclusion:**

The prevalence and infection sites of CNS differed from those of CPS. Genetic analyses indicated that genetic divergence, possible zoonotic transfer of MRSA, and the involvement of *sec *as important virulence factors for of *S. aureus *that lead to mastitis in goats.

## Background

Important pathogenic species of coagulase-positive and negative *Staphylococcus *(CPS and CNS, respectively) are *S. aureus *and *S. epidermidis*, respectively, which are frequently isolated from the environment and infected animals and humans [1,2]. *S. aureus *can cause severe blood infections and necrotizing fasciitis in humans, wound infection and mastitis in cattle, horses, pigs, and goats, exudative epidermitis in pigs, pyoderma in horses, dogs, and cats, pyemic sheep [2,3]. As an opportunistic pathogen, CNS occasionally cause subclinical or clinical mastitis. For example, in cattle mastitis can be caused by *S. capitis, S. chromogenes, S. cohnii, S. epidermidis, S. haemolyticus, S. hominis, S. simulans, S. warneri, S. hyicus*, and *S. caseolyticus*; in goats by *S. caprae*; and in cattle and sheep by *S. xylosus *[2]. However, CNS can damage breast tissue to increase the somatic cell counts and decrease milk quality and production [4]. The identification of CNS species is essential to determine their pathogenicity and to develop management practices to prevent mastitis. Due to difficult and expensive procedures for identifying these organisms, many laboratories do not perform these assays.

As opportunistic pathogens, *S. aureus *and *S. epidermidis *can also cause bacteremia, subacute endocarditis, and can form biofilms on heart valve prostheses, shunts, total joint arthroplasty or surgical sutures in humans [5]. Some *S. aureus *isolates possess a staphylococcal enterotoxin (SE), which is a virulence factor for foodborne disease and can form several products from SE genes, designates *sea *- *seu *[6,7]. These SE genes are associated with host-specific infection, such as *seb *for foodborne infection in humans, *sea *for bovine infection [8], and *sec *for mastitis in goats and bovine [9]. However, more than 10 SE genes were identified in isolates obtained from goats [7]. As the most prevalent pathogen in both hospitals and communities, *S. aureus *can become methicillin (oxacillin)-resistant *S. aureus *(MRSA/ORSA) by the introduction of an exogenous mobile staphylococcal chromosomal cassette, *mec *(SCC*mec*), encoding a low-affinity penicillin-binding protein 2a responsible for methicillin/oxacillin resistance [10-14]. Based on variations in the *ccr *operon and *mec *complexes, at least seven SCC*mec *types have been identified [14-16]. Moreover, methicillin-resistant CNS has been identified in animals [17].

The extensive use of antibiotics in hospitals and animals has increased the emergence of MDR *Staphylococcus *[1]. Since the emergence of MRSA isolated from humans in the 1960s [18], MRSA has been frequently isolated in hospitals, dogs, and horses as a zoonotic pathogen [17,19,20]. Our recent study characterized MDR *S. aureus *from dairy goats in 2006-2007 and no MRSA was isolated [21]. The current study investigated the prevalent *Staphylococcus *spp. and characterized the MDR-*S. aureus *and MRSA isolates collected from goats in 2008 by nucleotide sequencing, pulse-field gel electrophoresis (PFGE) analysis and polymerase chain reaction (PCR) analysis to elucidate the origins of MRSA isolates and the SE types associated with mastitis in goats.

## Results

### Identification of *staphylococcus *species

In total, 555 samples were collected from six bodily regions of dairy goats and three environmental sources in 2008. Among 45.4% (252/555) samples with bacterial growth on blood agar, 137 staphylococcal strains and 115 non-staphylococcal strains were identified. Five major *Staphylococcus *spp. were identified and listed in decreasing order of isolate number: *S. lentus *(31), *S. aureus *(27), *S. epidermidis *(23), *S. xylosus *(17) and *S. caprae *(3), and others (36).

### Prevalent *staphylococcus *species in different sampling locations and farms

Staphylococcal species were frequently isolated from milk, anus, vagina, nasal cavity, and udders (Table 1). However, the predominant infection sites differed between *S. aureus *and CNS. On average, the prevalence of *S. aureus *was 18.9 and 0.7% for goats and environmental samples, respectively, and differed among farms and body regions of goats (Table 1). In contrast to being absent in the anus and dorsum, *S. aureus *was predominant in the nasal cavity (12), vagina (9), and milk (4); in addition, CNS was prevalent in the milk (54), vagina (26), and anus (26).The highest prevalence of *S. aureus *was found in Farm A, followed by Farms D, C and B. In contrast, the highest prevalence of CNS was found in goats on Farm D followed by Farms C, B, and A.

**Table 1:** Prevalence of *Staphylococcus *spp. in different goat farms

Sample site	Farm A (%)		Farm B (%)		Farm C (%)		Farm D (%)		Total (%)	SA (%)	CNS^b^ (%)
				
	SA	CNS	SA	CNS	SA	CNS	SA	CNS			
Dorsum	0 (0/21)	0 (0/21)	0 (0/21)	0 (0/21)	0 (0/20)	0 (0/20)	0 (0/11)	0 (0/11)	0 (0/73)	0 (0/137)^a^	0 (0/137)
Vagina	10.0 (2/20)	10.0 (2/20)	9.5 (2/21)	19.0 (4/21)	15.0 (3/20)	25.0 (5/20)	18.2 (2/11)	45.5 (5/11)	34.7 (25/72)	6.6 (9/137)	12.4 (16/137)
Anus	0 (0/20)	20.0 (4/20)	0 (0/21)	38.1 (8/21)	0 (0/20)	45.0 (9/20)	0 (0/11)	45.5 (5/11)	36.1 (26/72)	0 (0/137)	19.0 (26/137)
Udder	0 (0/20)	15.0 (3/20)	0 (0/21)	9.5 (2/21)	5.0 (1/20)	5.0 (1/20)	0 (0/11)	9.1 (1/11)	11.1 (8/72)	0.7 (1/137)	5.1 (7/137)
Nasal cavity	35.0 (7/20)	0 (0/20)	9.5 (2/21)	9.5 (2/21)	5.0 (1/20)	10.0 (2/20)	18.2 (2/11)	36.4 (4/11)	27.8 (20/72)	8.8 (12/137)	5.8 (8/137)
Milk	2.5 (1/40)	20.0 (8/40)	2.4 (1/42)	33.3 (14/42)	2.5 (1/40)	42.5 (17/40)	4.3 (1/23)	52.2 (12/23)	37.9 (55/145)	2.9 (4/137)	39.4 (51/137)
Apparatus	0 (0/15)	6.7 (1/15)	0 (0/8)	0 (0/8)	0 (0/6)	0 (0/6)	0 (0/5)	0 (0/5)	2.9 (1/34)	0 (0/137)	0.7 (1/137)
Bulk tank	50.0 (1/2)	0 (0/2)	0 (0/2)	0 (0/2)	0 (0/2)	50.0 (1/2)			33.3 (2/6)	0.7 (1/137)	0.7 (1/137)
Water	0 (0/3)	0 (0/3)	0 (0/2)	0 (0/2)	0 (0/2)	0 (0/2)	0 (0/2)	0 (0/2)	0 (0/9)	0 (0/137)	0 (0/137)

Total	6.8 (11/161)	11.2 (18/161)	3.1 (5/159)	18.9 (30/159)	4.0 (6/150)	23.3 (35/150)	5.9 (5/85)	31.8 (27/85)	24.7 (137/555)	19.7 (27/137)	80.3 (110/137)

^a^137 isolates consisted of 27 *S. aureus *isolates and 110 CNS isolates

^b^CNS: catalase-negative *Staphylococcus *spp

### Antimicrobial susceptibility

Fifteen antimicrobial agents were used to characterize *S. aureus *strains and could be divided into 13 antibiograms. Nearly all isolates were susceptible to enrofloxacin, gentamycin, neomycin, and vancomycin; in addition, over 60% of the isolates were also resistant to penicillin (P), ampicillin (A), cephalothin, tetracycline (T), and oxytetracycline (Ot) (Table 2). In this study, 11 (40.7%) MRSA isolates were identified in nasal cavity, vagina, and goat milk only. Isolates from the nasal cavity exhibited the highest antimicrobial resistance. Compared with the antimicrobial resistance found for isolates identified in 2006-2007, isolates from 2008 revealed an increase in resistance to ampicillin, bacitracin (B), cloxacin (Cl), oxacillin (Ox), penicillin G, and streptomycin (S) (Table 2). Although 88.9% of isolates were resistant to more than one antimicrobial agent; the most prevalent antibiograms were AOtPT (29.6%, 8/27) and ABClOtOxPST (25.9%, 7/27). The antibiogram number varied among farms from six in Farm A to five in Farm B, four in Farm D and two in Farm C. Differences in antibiograms and their number may indicate the different bacterial sources in each farm. The minimum inhibitory concentrations (MIC) of 11 MRSA isolates to methicillin ranged from 16 to 256 μg/mL, and varied among farms and bodily region. The highest MIC was found in isolates collected from the nasal cavity (64-256 μg/mL), followed by goat milk (64 μg/mL), and vagina (16-64 μg/mL).

**Table 2:** Susceptibility to 15 antimicrobials for *Staphylococcus aureus *isolated from the goat nasal cavity, vagina, and milk

Antimicrobial	2006-2007	Location (2008)			
		
		Total	Goat milk	Nasal cavity	Vagina
Penicillin G	57.0 (49/86)	85.2 (23/27)	50.0 (1/2)	100 (12/12)	88.9 (8/9)
Ampicillin	57.0 (49/86)	81.5 (22/27)	100 (2/2)	100 (12/12)	66.7 (6/9)
Bacitracin	0 (0/86)	29.6 (8/27)	0 (0/2)	50.0 (6/12)	22.2 (2/9)
Cephalothin	0 (0/86)	70.4 (19/27)	50 (1/2)	66.7 (8/12)	77.8 (7/9)
Furoxime	0 (0/86)	14.8 (4/27)	50.0 (1/2)	8.3 (1/12)	11.1 (1/9)
Cloxacillin	1.2 (1/86)	48.1 (13/27)	50.0 (1/2)	58.3 (7/12)	44.4 (4/9)
Enrofloxacin	2.3 (2/86)	0 (0/27)	0 (0/2)	0 (0/12)	0 (0/9)
Gentamicin	14.0 (12/86)	7.4 (2/27)	50.0 (1/2)	0 (0/12)	11.1 (1/9)
Neomycin	10.5 (9/86)	7.4 (2/27)	50.0 (1/2)	0 (0/12)	11.1 (1/9)
Oxacillin	0 (0/86)	40.7 (11/27)	50.0 (1/2)	58.3 (7/12)	33.3 (3/9)
Oxytetracycline	72.1 (62/86)	66.7 (18/27)	100 (2/2)	83.3 (10/12)	44.4 (4/9)
Streptomycin	22.1 (19/86)	40.7 (11/27)	50.0 (1/2)	41.7 (5/12)	44.4 (4/9)
Tetracycline	69.8 (60/86)	74.1 (20/27)	100 (2/2)	83.3 (10/12)	66.7 (6/9)
Sulfamethoxazole/Trimethoprim	2.3 (2/86)	7.4 (2/27)	50.0 (1/2)	8.3 (1/12)	0 (0/9)

### Staphylococcal enterotoxin types

Of the nine enterotoxin genes examined, only *sec *and *see *were identified in *S. aureus *isolates and no SE genes were found in 71 CNS strains (Table 3). Eight out of 10 *S. aureus *strains isolated from milk of 15 goats with mastitis were shown to possess *sec*. PCR-restriction fragment length polymorphism (RFLP) analysis of *sec *PCR products revealed an identical *Alu*I restriction pattern for all strains. In 27 *S. aureus *bovine strains, only *sea *was identified in three strains.

**Table 3:** Prevalence of enterotoxin genes in different *Staphylococcus *spp

Enterotoxin genes	Positive rate (%)			
	
	Goat			Bovine
		
	*S. aureus *(n = 113)^a^	*S. aureus *(n = 10)^b^	CNS (n = 71)	*S. aureus *(n = 27)^c^
*sea*	0 (0/113)	0 (0/10)	0 (0/71)	11.1 (3/27)
*seb*	0 (0/113)	0 (0/10)	0 (0/71)	0 (0/27)
*sec*	22.1 (25/113)	80.0 (8/10)	0 (0/71)	0 (0/27)
*sed*	0 (0/113)	0 (0/10)	0 (0/71)	0 (0/27)
*see*	0.9 (1/113)	0 (0/10)	0 (0/71)	0 (0/27)
*seg*	0 (0/113)	0 (0/10)	0 (0/71)	0 (0/27)
*seh*	0 (0/113)	0 (0/10)	0 (0/71)	0 (0/27)
*sei*	0 (0/113)	0 (0/10)	0 (0/71)	0 (0/27)
*sej*	0 (0/113)	0 (0/10)	0 (0/71)	0 (0/27)

^a^113 strains comprised 27 strains in 2008 and 86 strains in 2006-2007

^b^10 *S. aureus *strains isolated from 15 goat mastitis samples

^c^27 *S. aureus *strains isolated from cattle with mastitis

### Characterization of oxacillin-resistant *S. aureus*

**Figure 1:**
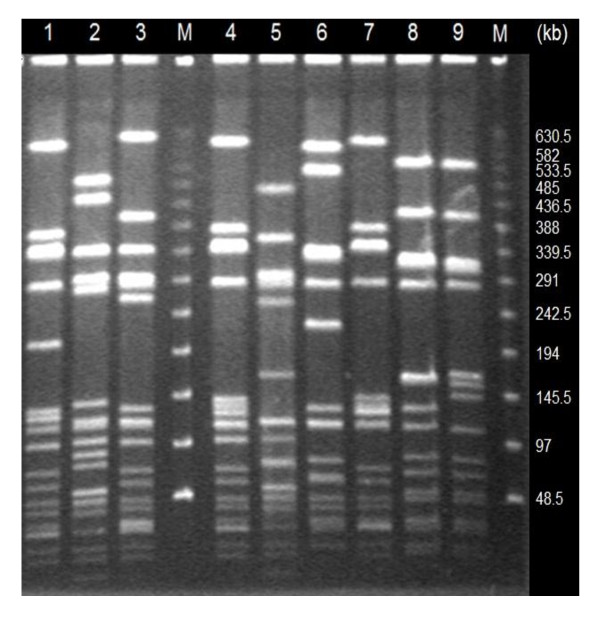
**PFGE patterns of *Sma*I-digested DNA isolated from clinical *S. aureus *goat isolates**. Lanes 1, 4 and 7: pulsotype I; lane 2: pulsotype II, lane 3: pulsotype III, lane 5: pulsotype IV, lane 6: pulsotype V; lane 8: pulsotype VIA; and lane 9: pulsotype VIB. M: λ DNA marker.

**Figure 2:**
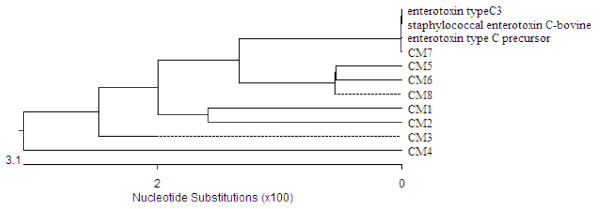
**Phylogenetic tree of *sec *genes from eight *Staphylococcus aureus *isolates from goats with mastitis**. The tree was constructed using the Megalign program of the Lasergene software. Scale bar represents number of nucleotide substitutions per? bases.

PCR amplification of 11 MRSA isolates identified SCC*mec *type III in nine isolates from Farms A (8) and B (1), and SCC*mec *type II in two isolates found in Farms B (1) and D (1) (Table 4). Two isolates could not be pulsotyped and six pulsotypes were determined (Figure 1). Four pulsotypes were identified in six isolates of Farm A, revealing identical patterns as pulsotypes A, C, and D in human isolates previously identified in Taiwan [22]. The remaining isolates belonged to pulsotypes VIA and VIB (no similar pulsotypes as human isolates) in Farm B and pulsotype V (pulsotype A of human isolate) in Farm D. Multi-locus sequence typing (MLST) analysis indicated that two isolates for which no pulsotype could be determined were ST59, the common MLST types of human isolates. Phylogenetic analysis of *sec *showed that sequence similarity ranged from 94.8-100% (Figure 2).

**Table 4:** Characterization of 11 oxacillin-resistant *S.aureus *strains

Strains	*mec*A	SCC*mec *type	Pulsotype^a^	Farms^b^	Pulsotype of human strain^c^
N0119	+	III	ND (ST59)^d^	A	
N0207	+	III	ND (ST59)	A	
N0229	+	III	I	A	A
N0262	+	III	II	A	C
N6292	+	III	III	A	A
N6294	+	III	I	A	A
V34	+	III	IV	A	D
V6292	+	III	I	A	A
V17	+	III	VIA	B	
N9	+	II	VIB	B	
MR28	+	II	V	D	A

^a^ND: DNA could not be digested by restriction enzyme *Sma*I

^b^Letters represent farms where samples were acquired

^c^Pulsotypes were determined as previously reported by Huang et al. [22]

^d ^ST determined based on the MLST website http://saureus.mlst.net/

## Discussion

CNS strains *S. lentus, S. epidermidis, S. xylosus*, and *S. caprae*, and *S. aureus *were identified as the predominant species infecting goats in this study. *S. aureus *and CNS differed in prevalence and bodily location in goats (Table 1). Additionally, CNS and *S. aureus *accounted for 38.2 and 11% of infections, respectively, in the milk of goats with mastitis [23], and 2.6% (35/1,372) and 17.3% (237/1,372), respectively, in the milk of dairy cows and goats in Taiwan [24]. In animals, the prevalence of *S. aureus *differed among countries, such as 2.2% in the United Kingdom [25], 6.7% in Canada [26], 9.1% in USA [27], 24.2% in Ethiopia [28], 29.6% in Korea [29], and 32.9% in Uruguay [30]. However, *S. aureus *accounts for 18.7% (64/342) of staphylococcal infections in goats [7]. In Taiwan, *S. aureus *infection in goats increased from 1.7% (12/706) [24] up to 2.5% (86/3,427) [31] and 4.9% (27/555) in this study. Sanitation may be another important factor that leads to an increase in *S. aureus *infection. Indeed, sanitation could reduce the *S. aureus *infection from 6.6 to 0.6% or from 14.6 to 3.0% [32,33]. In Taiwan, *S. aureus *infection in milk ranged from 0-5.2% among farms [21]. In the present study, the prevalence ranged from 3.1 to 6.8% for *S. aureus *and from 11.2 to 31.8% for CNS among farms, suggesting that CNS infection is more common in goats.

Early reports indicated that staphylococcal infections in goat bodily regions differed and ranged from 6.1% (21/342) in the armpits up to 70.8% (242/342) on the skin of udders and mamilla, and contributed to only 0.7% contamination of the apparatus [7]. Although lacking statistical analysis in this study, *Staphylococcus *was identified in 42.3% (58/137) and 5.8% (8/137) of milk and udders, respectively (Table 1), suggesting that *Staphylococcus *is a major bacterial cause of mastitis in goats that can then infect humans through unsanitary milk. As an important virulence factor causing foodborne disease in humans, predominant SE genes are associated with outbreaks in certain countries, such as *sea *in France [34], *seb *in eastern Slovakia, Tehran, and Japan [35-37], *sec*-2 and *sec*-3 in Taiwan [38]. Furthermore, particular *sec *types is also associated with food-related *Staphylococcus *spp. [39], in *S. aureus*, and CNS isolated from the milk of sheep [7,40], and in *S. aureus *collected from sheep or cows with mastitis [41]. Additionally, *sea *was determined to be the major type in *S. aureus *from cows with mastitis [8]. As shown in Table 3, *sec *and *sea *were the major SE genotypes found in goats and cows, respectively, and *sec *was associated with mastitis in goats. These data confirmed the importance of the *sec *gene involved in goat mastitis pathogenesis [42]. In cattle, the SEC toxin (not toxic shock syndrome toxin type 1 or TSST-1) was previously shown to significantly increase somatic cell counts and enhance the severity of the mastitis in acute mastitis [43]. Although SE genes have been found in CNS species, such as *S. xylosus, S. warneri*, and *S. chromogenes *isolated from cows and goats [7,40,44,45], they were only identified in CPS but not CNS isolated previously in Taiwan [46,47] and in the present study (Table 3). As an opportunistic pathogen that can cause mastitis in cattle and goats [1,21,48,49], *S. aureus *infection can also cause clinical symptoms in cattle [50] but is typically asymptomatic in goats [21,48].

Using penicillin, ampicillin, and tetracycline antibiotics to treat bacterial infections in animals often increases resistance to these antibiotics. In cattle, *S. aureus *or other *Staphylococcus *spp. causing mastitis were more resistance to penicillin and ampicillin, streptomycin, tetracycline, and oxytetracycline [1,24,49,51]. Our data showed an increased resistance to penicillin, ampicillin, cloxacillin, and cephalothin from 2006 to 2008, in addition to the appearance of MRSA isolates from goats in 2008 (Table 2). Furthermore, the MRSA isolates identified in this study belonged to the major nosocomial SCC*mec *types: SCC*mec *type II and III [52,53]. Zoonotic transfer of MRSA has been reported between horses and humans in the USA [20,54], between cattle and humans in Korea [55], and between livestock and humans in Taiwan [56,57]. Our data indicate that MRSA isolates may have been acquired from humans or transmitted from different goat breeding farms.

PFGE analysis is typically performed to trace the pathogens responsible for outbreaks. Containing a thick cell wall, *S. aureus *must be treated with lysostaphin, not lysozyme, to break the pentaglycine linkage within the peptidoglycan [58,59]. For genomic analysis of *S. aureus*, genomic DNA cannot be digested by restriction enzyme *Sma*I and diverse genomic variations in size [60] limit the utility of PFGE analysis. However, pulsotypes appear to correlate with human disease. Pulsotype D *S. aureus *is associated with more severe symptoms than pulsotype type C bacteria that only cause mild symptoms [50]. In Taiwan, MRSA accounts for 53-83% of *S. aureus *isolates from hospitals [61] and the major pulsotypes of human MRSA are pulsotype A, followed by types C and D [22]. In this study, pulsotype A was also the most prevalent type of goat MRSA isolate (Figure 1, Table 4). Additionally, PFGE analysis also revealed diverse sources of MRSA in Farm A and a single origin in Farms B and D (Table 4).

## Conclusion

The current study was the first report of the appearance of MRSA strains and *sec*-associated mastitis in goats from Taiwan. Analysis of SCC*mec *types and pulsotypes revealed that the genetically diverse MRSA strains might have been acquired from humans or transferred from different goat breeding farms.

## Methods

### Source of bacterial isolates

Samples were acquired aseptically according to the procedure described by the National Mastitis Council (NMC) with some modification [62]. Informed consent was obtained from all farm owners prior to the start of this study. A total of 555 samples were collected from the milk, anus, dorsum, nasal cavity, udders, and vagina of goats, in addition to milking apparatus, bulk tank, and water of environments at four different goat farms in Taiwan during 2008. Isolates (n = 137) were characterized by culture on blood agar [63], morphology, Gram stain, catalase test, and growth on *Staphylococcus *medium No. 110 (Oxoid, Basingstoke, UK). Furthermore, *Staphylococcus *species were biotyped according to method of Myllys et al. [64] using the API Staph identification kit (BioMerieux, Marcy l'Etoile, France). Additionally, 86 isolates collected in 2006-2007 were compared with those isolated in 2008. CPS (BCRC 14958, ATCC 27664) and CNS (BCRC 15228) strains were purchased from the Bioresource Collection and Research Center (BCRC, Taiwan). Twenty-seven *S. aureus *strains isolated from cattle with mastitis were kindly provided by Professor Shih-Te Chuang, (College of Veterinary Medicine, National Chung Hsing University, Taiwan) for examination of enterotoxin genotypes. Human isolates were kindly provided by Professor Chishih Chu, (the Department of Microbiology, Immunology, and Biopharmaceuticals, National Chiayi University). The use of all bacteria was supervised by the Biological Security Committee of National Chiayi University in accordance with the laws of Taiwan. The protocols and regulations used during the study were performed according to the guidelines of"Animal Use Protocol, the Institutional Animal Care and Use Committee (IACUC), National Chiayi University" with great care for animal welfare and adherence to the law.

### Antimicrobial susceptibility test

The disc diffusion method and the guidelines of the Clinical and Laboratory Standards Institute (CLSI) standards and the manufacturer were used to determine the susceptibility of each isolate to ampicillin (AMP; 10 μg), bacitracin (BAC; 10 units), oxacillin (OXA; 1 μg), cefuroxime (CXM; 30 μg), cephalothin (CEP; 30 μg), cloxacillin (CLO; 5 μg), enrofloxacin (ENR; 5 μg), gentamicin (GEN; 10 μg), neomycin (NEO; 30 μg), oxytetracycline (OXY; 30 μg), penicillin G (PEN; 10 U), streptomycin (STR; 10 μg), sulfamethoxazole/trimethoprim (Sxt; 23.75 μg for S and 1.25 μg for t), tetracycline (TET; 30 μg), and vancomycin (VAN; 30 μg) [65]. Results of the antimicrobial susceptibility were also validated using *Escherichia coli *(ATCC No. 25922). Bacto discs were purchased from Becton Dickinson (Sparks, MD, USA). Finally, MIC to oxacillin of each ORSA isolate was determined by Etest (AB Biodisk, Solna, Sweden).

### SCC*mec *typing

DNA templates of MRSA isolates were prepared for PCR amplification using the QIAamp DNA Mini Kit (Qiagen, Valencia, CA, USA). Table 5 lists the primers used to identify *mecA *and the SCC*mec *types [52,66,67]. A 50-μL PCR reagent included 5 μL of DNA template, 20 μM of each primer, 0.2 mM dNTPs, 5 μL of 10× PCR reaction buffer, 1.4 U Taq DNA polymerase, and 33.3 μL distilled water. The PCR conditions were a initial denaturation at 94°C for 4 min, followed by 30 cycles of 94°C for 30 s, 53°C for 30 s, and 72°C for 1 min and a final extension at 72°C for 4 min. PCR products were separated by 2% agarose at 50 V for 1 hr and visualized under ultraviolet illumination after ethidium bromide (EtBr) staining.

**Table 5:** Primers used for amplification of SCC*mec *type and enterotoxin gene

Locus	Primer	Oligonucleotide sequence (5'-3')	Amplicon size (bp)	SCC*mec *type^e^	Reference
SCC*mec*					

A	CIF2 F2 ^a^	TTCGAGTTGCTGATGAAGAAGG	495	I	[17]
	CIF2 R2 ^a^	ATTTACCACAAGGACTACCAGC			

B	KDP F1 ^b^	AATCATCTGCCATTGGTGATGC	284	II	[18]
	KDP R1 ^b^	CGAATGAAGTGAAAGAAAGTGG			
C	MECI P2 ^b^	ATCAAGACTTGCATTCAGGC	209	II, III	
	MECI P3 ^b^	GCGGTTTCAATTCACTTGTC			

D	DCS F2^a^	CATCCTATGATAGCTTGGTC	342	I, II, IV	[17]
	DCS R1^a^	CTAAATCATAGCCATGACCG			
E	RIF4 F3^c^	GTGATTGTTCGAGATATGTGG	243	III	[18]
	RIF4 R9^c^	CGCTTTATCTGTATCTATCGC			
F	RIF5 F10^c^	TTCTTAAGTACACGCTGAATCG	414	III	
	RIF5 R13^c^	GTCACAGTAATTCCATCAATGC			
G	IS431 P4^b^	CAGGTCTCTTCAGATCTACG	381		
	pUB110 R1^b^	GAGCCATAAACACCAATAGCC			
H	IS431 P4^c^	CAGGTCTCTTCAGATCTACG	303		
	pT181 R1^c^	GAAGAATGGGGAAAGCTTCAC			
*mecA*	MECA P4^d^	TCCAGATTACAACTTCACCAGG	162	Internal control	[16]
	MECA P7^d^	CCACTTCATATCTTGTAACG			

Staphylococcal enterotoxin (SEs)					
*sea*	ESA 1	ACGATCAATTTTTACAGC	544		[68]
	ESA 2	TGCATGTTTTCAGAGTTAATC			
*seb*	ESB 1	GAATGATATTAATTCGCATC	416		[69]
	ESB 2	TCTTTGTCGTAAGATAAACTTC			
*sec*	ESC 1	GACATAAAAGCTAGGAATTT	257		[70]
	ESC 2	AAATCGGATTAACATTATCCA			
*sed*	ESD 1	TTACTAGTTTGGTAATATCTCCTT	334		[71]
	ESD 2	CCACCATAACAATTAATGC			
*see*	ESE 1	ATAGATAAAGTTAAAACAAGCAA	170		[72]
	ESE 2	TAACTTACCGTGGACCC			
*seg*	ESG 1	ACGTCTCCACCTGTTGAAGG	400		[73]
	ESG 2	TGAGCCAGTGTCTTGCTTTG			
*seh*	ESH 1	TCACATCATATGCGAAAGCAG	357		[74]
	ESH 2	TAGCACCAATCACCCTTTCC			
*sei*	ESI 1	TGGAACAGGACAAGCTGAAA	467		[73]
	ESI 2	TAAAGTGGCCCCTCCATACA			
*sej*	ESJ 1	CAGCGATAGCAAAAATGAAACA	426		[75]
	ESJ 2	TCTAGCGGAACAACAGTTCTGA			
*Sec*^f^	setC-F setC-R	AGATTTAGCAAAGAAGTACAAAGATG AAGGTGGACTTCTATCTTCACACTT	490		[76]

^a^Relative to accession no. AB033763, SCC*mec *type I

^b^Relative to accession no. D86934, SCC*mec *type II

^c^Relative to accession no. AB037671, SCC*mec *type III

^d^Relative to accession no. Y00688, *mecA *gene.

^e^Loci G and H were included to distinguish variants IA from I and IIIA from III, respectively

^f^Primer pair used for PCR-RFLP

### PCR identification of SE types and PCR-RFLP analysis

Staphylococcal enterotoxin genes, *sea-sej*, were amplified by PCR using primers listed in Table 5[68-76]. 50-μL PCR reagent was described earlier. The cycling conditions for multiplex PCR were 5 min at 94°C for initial denaturation, followed by 30 cycles of 1 min at 94°C, 30 s at 60°C, 1 min at 72°C and a final extension at 72°C for 10 min to amplify *sea *- *see*. For amplification of *seg*-*sej*, initial denaturation was performed for 3 min at 94°C, followed by 30 cycles of 30 s at 94°C, 30 s at 60°C, and 30 s at 72°C and a final extension at 72°C for 10 min. PCR products of *sec *were purified from agarose gels using the Gene-Spin-V^2 ^Miniprep purification kit (Protech Technology, Solon, OH, USA), and the purified products were digested with restriction enzyme *Alu*I and separated by 1% agarose gel run at 50 V for 1 hr. Furthermore, the purified PCR products were sequenced and analyzed using the Megalign program of the Lasergene software (DNAstar, Madison, Wisconsin, USA).

### Genetic typing

The genotype of each MRSA isolate was determined by restriction enzyme *Sma*I-digested PFGE analysis, as previously described [77]. Briefly, whole-cell embedded agarose plugs were digested with *Sma*I and then resolved by a CHEF DR-III apparatus (Bio-Rad, Hercules, CA, USA). Finally, the MRSA isolate BCRC 15211 was used as the standard size marker. Pulsotypes and MLST types of MRSA were determined based on the method described by Enright et al. [78] and analysis of MLST databases, respectively.

## Abbreviations

AMP: ampicillin; BAC: bacitracin; CEP: cephalothin; CLO: cloxacillin; CNS: coagulase-negative *Staphylococcus; *CPS: coagulase-positive *Staphylococcus*; CXM: cefuroxime; ENR: enrofloxacin; EtBr: ethidium bromide; GEN: gentamicin; MDR: multi-drug resistance; MIC: minimum inhibitory concentration; MRSA/ORSA: methicillin/oxacillin-resistant *Staphylococcus aureus; *NEO: neomycin; OXA: oxacillin; OXY: oxytetracycline; PCR-RFLP: polymerase chain reaction- restriction fragment length polymorphism; PEN: penicillin G; PFGE: pulsed-field gel electrophoresis; SCC*mec*: staphylococcal cassette chromosome *mec; *SE: staphylococcal enterotoxin; STR: streptomycin; Sxt: sulfamethoxazole/trimethoprim; TET: tetracycline; VAN: vancomycin.

## Authors' contributions

CC instructed the molecular genetics and phylogenetic studies, and edited the manuscript. CYY designed experiments and assisted in data analysis. YHL performed the experiments. YCS coordinated and designed the experiments, and prepared the manuscript. All authors read and approved the final manuscript.

## Authors' information

Chishih Chu is a Professor in the Department of Microbiology, Immunology, and Biopharmaceuticals. Changyou Yu is a Professor in the Department of Veterinary Medicine. Yanhaui Lee was a Master student in the Department of Veterinary Medicine. Yaochi Su is an Associate Professor in the Department of Veterinary Medicine, National Chiayi University, Taiwan, Republic of China.
